# Differential Roles of Lipin1 and Lipin2 in the Hepatitis C Virus Replication Cycle

**DOI:** 10.3390/cells8111456

**Published:** 2019-11-18

**Authors:** Victoria Castro, Gema Calvo, Ginés Ávila-Pérez, Marlène Dreux, Pablo Gastaminza

**Affiliations:** 1 Department of Cellular and Molecular Biology Centro Nacional de Biotecnología-Consejo Superior de Investigaciones Científicas, Centro Nacional de Biotecnología-C.S.I.C., Calle Darwin 3, 28049 Madrid, Spain; vcastro@cnb.csic.es (V.C.); gcalvo@cnb.csic.es (G.C.); gines_ap@hotmail.com (G.Á.-P.); 2 CIRI, Inserm, U1111, Université Claude Bernard Lyon 1, CNRS, UMR5308, École Normale Supérieure de Lyon, Univ Lyon, F-69007 Lyon, France; marlene.dreux@ens-lyon.fr

**Keywords:** hepatitis C, lipid homeostasis, lipin, lipid signaling, Golgi apparatus

## Abstract

Although their origin, nature and structure are not identical, a common feature of positive-strand RNA viruses is their ability to subvert host lipids and intracellular membranes to generate replication and assembly complexes. Recently, lipin1, a cellular enzyme that converts phosphatidic acid into diacylglycerol, has been implicated in the formation of the membranous web that hosts hepatitis C virus (HCV) replicase. In the liver, lipin1 cooperates with lipin2 to maintain glycerolipid homeostasis. We extended our previous study of the lipin family on HCV infection, by determining the impact of the lipin2 silencing on viral replication. Our data reveal that lipin2 silencing interferes with HCV virion secretion at late stages of the infection, without significantly affecting viral replication or assembly. Moreover, uninfected lipin2-, but not lipin1-deficient cells display alterations in mitochondrial and Golgi apparatus morphology, suggesting that lipin2 contributes to the maintenance of the overall organelle architecture. Finally, our data suggest a broader function of lipin2 for replication of HCV and other RNA viruses, in contrast with the specific impact of lipin1 silencing on HCV replication. Overall, this study reveals distinctive functions of lipin1 and lipin2 in cells of hepatic origin, a context in which they are often considered functionally redundant.

## 1. Introduction

Hepatitis C virus (HCV) is a member of the Hepacivirus genus in the *Flaviviridae* family [1]. Virions are enveloped and carry a positive-strand RNA genome of approximately 9600 nucleotides. The viral genome encodes a unique polyprotein that is processed co- and post-translationally to produce 10 major viral proteins [2]. The three major structural viral components of the virion include core protein, that encapsidates the viral genome and E1/E2 glycoprotein complexes that mediate virus entry [3]. Non-structural proteins NS3, NS4A, NS5A and NS5B are sufficient to build membranous viral replication complexes in infected host cells [4,5]. NS2 and p7 coordinate infectious particle assembly, which is coupled with viral RNA replication and mediated by structural proteins [6,7].

Compelling evidence indicates a strong interference of HCV infection with host cell lipid metabolism [8]. This is manifested by the reliance of virtually all steps in the viral lifecycle on host factors involved in lipid metabolism [9,10]. In fact, HCV virions are chimeric structures carrying host apolipoproteins, cholesterol and triglycerides, in addition to viral structural proteins [11,12,13]. These host components determine HCV virion recognition by cellular receptors and also facilitate immune evasion by their resemblance to hepatic lipoproteins [14]. While host components mediate initial attachment of the virions to the cell surface, E1/E2 complexes are recognized by host receptors to trigger subsequent steps in particle internalization by clathrin-mediated endocytosis that result in E1/E2-mediated membrane fusion and delivery of the viral genome to the cytoplasm [6]. Translation of the incoming genomes into viral proteins triggers recruitment of host factors, that are essential for remodeling of cellular membranes into a characteristic membranous web (MW) of vesicles and associated cytoplasmic lipid droplets (LD) [15]. Viral protein expression deeply transforms the cytoplasm of infected cells, promoting the proliferation of membranous compartments associated with viral RNA replication in the form of double and multiple membrane vesicles (DMVs; MMVs) [5,16]. MW formation facilitate functional and physical association of DMVs to enlarged cytoplasmic lipid droplets to coordinate infectious virion assembly [7,10,17]. Virus assembly relies on several factors involved in the production of hepatic lipoproteins, such as apoB, apoE, DGAT1 or MTP [13,18,19,20]. Once assembled, infectious virus precursors are secreted to the extracellular milieu through a pathway that co-opts host vesicular transport and depends on endosomal components [21,22,23,24,25]. During and after secretion, extracellular infectious virions acquire characteristics of very low-density lipoproteins (VLDL), such as incorporation of host apoproteins apoE, apoA1, apoB and triglycerides [11,12,13,26,27,28].

We have recently shown that lipin1, a key enzyme in glycerophospholipid biosynthesis, is rate limiting for the formation of HCV-induced membranous compartments and subsequent HCV RNA replication [29]. Lipin1 is the best characterized member of lipins, a family of phosphatidate phosphatase (PAP) enzymes, which catalyze the conversion of phosphatidic acid (PA) to diacylglycerol (DAG) not only in the glycerol-3-phosphate (G3P) glycerophospholipid biosynthetic pathway [30], but also using discrete PA pools generated by specialized membrane phospholipases as substrate [31,32]. Three different genes encoding lipins (LPIN1, LPIN2 and LPIN3) have been described in mammals [33]. Although the encoded proteins (lipin1, lipin2 and lipin3) only display partial sequence homology, they share two conserved domains at the N and C-terminus of the protein, denominated N-LIP and C-LIP [34]. The salient characteristics of this family that differentiate them from other lipid phosphatases are: i) their enzymatic activity requires Mg^2+^; ii) they display a strong specificity for PA as substrate and iii) they are not constitutively associated with membranes [35,36]. All lipins display PAP activity, being lipin1 the one displaying the highest specific activity [34]. Lipin1 and lipin2 display dual functions as PAP enzyme at ER membranes and as transcriptional coactivator in the nucleus, although the functional relevance of lipin2 transcriptional coactivation activity is still unclear [37]. Lipin PAP activity can be regulated by phosphorylation, mainly by mTOR, depending on metabolic and homeostatic needs of the cell [38]. Although lipin2 and lipin3 are also phosphorylated, the phosphorylation status appears to influence only lipin1 PAP activity [39,40].

In humans, lipin1 is predominantly expressed in muscle, nerve and testis, while lipin2 is abundant in liver and small intestine. Lipin3, the least studied member of the family is relatively more abundant in skin and small intestine [37]. LPIN1 loss-of-function is genetically associated with rhadbomyolisis [41], while loss of lipin2 protein expression is associated with Majeed syndrome, a rare inflammatory disease [42]. In the liver, lipin1 and lipin2 coordinate lipid homeostasis. LPIN1 or LPIN2- deficient mice display a relatively normal glycerolipid metabolism, while double knock-out mice are embryonically lethal [43]. In these models, lack of lipin1 expression is compensated by enhanced accumulation of lipin2 and *vice versa*. This, together with the fact that none of the diseases genetically associated with LPIN1 or LPIN2 are strongly manifested as alterations of liver metabolism in adults, indicate that one may functionally compensate essential aspects of the liver lipid metabolism in the absence of the other [43].

Our virological studies suggest a non-specific role of lipin2 in the replication of different *Flaviviridae*, which contrasts with the specific impact of lipin1 silencing on HCV replication. Study of different aspects of the virus lifecycle reveal that lipin2 silencing interferes with virion secretion, an aspect of the infection that is not altered by lipin1 silencing. Moreover, we have documented a strong impact of lipin2, but not lipin1, silencing on Golgi apparatus and mitochondrial morphology, revealing divergent roles in lipin1 and lipin2 in cells of hepatic origin, a context in which they are often considered functionally redundant.

## 2. Materials and Methods

### 2.1. Cells and Viruses

Huh-7 human hepatoma cells [44], Huh-7.5.1 [45] and HEK293T cells [46] were cultured in Dulbecco’s Modified Eagles’s Medium supplemented with 100 mM HEPES, non-essential aminoacids (Sigma-Aldrich, Dorset, UK) and Penicillin/Streptomycin (Sigma-Aldrich) and 10% Fetal Calf Serum as previously described [29]. Huh-7 cells constitutively expressing PKC-C1-GFP or YFP-tagged mitochondria were produced by transfection of plasmid pEF-BOS-C1-PKCθ kindly provided by Isabel Mérida (CNB-CSIC, Madrid, Spain) and mito-YFP (Clontech, Palo Alto, CA, USA). Stably transfected cell populations were selected by supplementing growth media with 250 µg/mL of G418 and subsequent selection of fluorescent cell populations by FACS. JFH-1 virus strain and a cell culture adapted JFH-1 variant D183 virus (D183v) have been described previously [45,47]. Viral stocks of the recombinant DENV (New Guinea C strain) [48] or ZIKV (BeH819015 strain) [49] viruses were produced by electroporation of the in vitro transcribed RNA genome as described previously [50,51]

### 2.2. Silencing Experiments

HIV-based lentiviral particles were produced by plasmid co-transfection as previously described [52]. Control as well as LPIN1-targeting vectors have previously been described [29,53]. LPIN2-targeting shRNA-expressing plasmids are commercially available (MISSION shRNA; Sigma-Aldrich) and target the following sequences CCTGATGATATTTACCTTGAT (shLPIN2-1) and CCCTGACTGATTTCAGCCATT (shLPIN2-1). Lentiviral vectors expressing control LPIN1 or LPIN2-specific shRNAs were used to inoculate Huh-7 cells. Twenty-four hours later, culture media was supplemented with 2.5 µg/mL of puromycin to select transduced cells. Selected cell populations were cultured for 6 days in the presence of puromycin until lipin silencing was verified by western blot. Cell viability was determined by a thiazolyl blue tetrazolium blue (MTT) formazan formation assay [54] and biomass estimation by crystal violet staining [55].

### 2.3. Western-Blot

Total protein samples were prepared in Laemmli buffer and separated using polyacrylamide denaturing gel electrophoresis (SDS-PAGE). Proteins were subsequently transferred onto polyvinylidene difluoride PVDF membranes and incubated with 5% skim milk (lipins) or 3% bovine serum albumin (BSA) in phosphate-buffered saline (PBS)-0.25% Tween20 for 1 h at room temperature (RT). Primary antibodies against lipin1 (clone B-12; Santa Cruz, Dallas, TX, USA), lipin2 (H-160; Santa Cruz) or lipin2 (G7; Santa Cruz), actin (ab8226; Abcam, Cambridge, UK) were diluted in blocking buffer and incubated for 1 h (4 h for lipins) at RT. Membranes were subsequently washed three times for 20 min with PBS-0.25% Tween20. Horseradish peroxidase-conjugated secondary antibodies were incubated for 1 h at room temperature in PBS for anti-actin or 5% milk-PBS-0.25% Tween20 for anti-lipin antibodies. Next, membranes were washed three times for 20 min at room temperature. Protein bands were detected using chemiluminescence and exposure in a Chemidoc device (BioRad, Watford, UK). Specific bands were quantitated using the Fiji software version 2.0.0-rc-69/1.52p (https://imagej.net/Fiji) [56].

### 2.4. HCV Infection Experiments

For multiple cycle infection experiments, Huh-7 cells expressing different lentiviral constructs were plated in a 12-well plate at a density of 5 × 10^4^ cells/well (Corning, Lowell, MA, USA), incubated overnight and inoculated at MOI 0.01 (multiplicity of infection) with D183 virus stocks. Cells were split 1:3 at day 3 to maintain the cultures subconfluent. Samples of the supernatants were collected at day 3 and 5 post-inoculation. For single cycle infection experiments, 5 × 10^4^ cells/well were seeded in 12-well plates (Corning). The day after, cells were inoculated with D183 virus stocks at MOI 10 and incubated at 37 °C. Five hours later, inoculum was removed and cells were washed twice with warm PBS. Forty-eight hours later cells and supernatants were collected for HCV RNA quantitation by RT-qPCR and infectious virus titer analysis as previously described [45]. Intra- and extracellular HCV infectivity titers were determined using endpoint dilution and immunofluorescence microscopy using a monoclonal antibody against E2 protein [57], as previously described [58].

### 2.5. Other Infection Experiments

Zika (ZIKV) and dengue virus (DENV) propagation studies were carried out in multiple cycle infection experiments (MOI 0.01) in Huh-7 cells. Samples of the supernatants were collected at the indicated time points and viral progeny was estimated by extracellular infectivity titration assays and expressed as focus forming units per ml (FFU/mL). Infectivity titers were determined in the supernatants of infected cells by end-point dilution, inoculation of Huh-7.5.1 cells and immunofluorescence microscopy using specific antibodies against Flavivirus E protein (mAb 4G2; [59]) as previously described [50,51].

### 2.6. Analysis of Viral Entry Using Retroviral Pseudotypes

Retroviral pseudotyped particles bearing viral envelope glycoproteins from HCV (HCVpp), vesicular stomatitis virus (VSVpp) or endogenous feline retrovirus RD114 (RD114pp) were produced in HEK293T cells as previously described [60,61,62]. Target Huh-7 cells (2 × 10^4^ cells per well) were plated on a 96-well plate. The next day, the cells were inoculated with retroviral particle preparations diluted to produce similar luciferase activity. Forty-eight hours later, cells were lysed and luciferase activity was detected and quantitated in a luminometer using a commercial kit (Luciferase Assay Kit; Promega, Madison, WI, USA). Relative infection values were calculated as percentage of the control. Hydroxyzine pamoate (5 µM; Sigma-Aldrich) was used in control cells as positive control of selective HCV entry inhibition [63].

### 2.7. In Vitro Transcription and HCV RNA Transfection

Plasmids containing the sequence corresponding to subgenomic JFH-1 replicons bearing a luciferase reporter gene have previously been described [64]. After digestion with the restriction enzyme MluI, the linearized plasmids were in vitro transcribed using a commercial kit (Megascript T7; Ambion, Paisley, UK). The resulting products were digested with DNAse and precipitated with LiCl. Pelleted RNA was washed with 75% and 100% ethanol, and resuspended in nuclease-free water. In vitro transcribed RNA was transfected with Lipofectamine 2000 (Life Technologies, Carlsbad, CA, USA) using the manufacturer´s recommendations. Luciferase activity was measured using a commercial kit (Dual Luciferase Assay System; Promega) at different times post-transfection. HCV polymerase inhibitor sofosbuvir (SFB; MedChem Express, Sollentuna, Sweden) was used as control [65].

### 2.8. Gaussia Secretion Experiments

Control, LPIN1kd and LPIN2kd cells (2.5 × 10^4^ cells/well) were transfected (Lipofectamine 2000; ThermoFisher, Waltham, MA, USA) in suspension in 96-well plates with a plasmid (pCAGGS-Gluc; 25 ng/well) transiently expressing secreted *Gaussia* luciferase [66]. The day after media was removed, replaced with fresh media and incubated for 8 h. Control cells were treated with 1 µg/mL of brefeldin A (BFA) as positive secretion inhibition control [67]. Samples of supernatant and cells were lysed and secretory activity was determined by the ratio of extracellular and intracellular luciferase activity as determined using a commercially available kit (New England Biolabs, Ipswich, MA, USA).

### 2.9. Confocal Immunofluorescence Microscopy

Sample preparation is identical to that described in Mingorance et al. [29], using commercially available antibodies against lipin1 (5195; Cell Signaling, Leiden, The Netherlands), lipin2 (H-160; Santa Cruz), Golgi-resident protein giantin (9B6 clone, Abcam), endoplasmic reticulum marker protein disulfide isomerase (PDI) subunit B (P4HB; 1D3 clone, Enzo Life Sciences, Exeter, UK) and the mitochondrial matrix protein aconitase 2 (ACO2; 6F12BD9 clone, Abcam). Confocal microscopy was performed with a Leica TCS SP5 laser scanning system (Leica Microsystems, Nussloch, Germany). Images of 1024 × 1024 pixels at 8-bit gray scale depth were acquired sequentially every 0.13–0.3 μM through a 63×/1.40 N.A. immersion oil lens (63×/1.20 N.A water immersion for in vivo mitochondria studies), employing LAS AF v 2.6.0 software (Leica Microsystems). For in vivo imaging cells were plated the day before onto a chambered coverslip (IBIDI, Gräfelfing, Germany) and labeled with Mitotracker^TM^ Red FM (ThermoFisher) as indicated by the manufacturer. During imaging, cells were maintained under controlled temperature (37 °C), CO_2_ (5%) and humidity (85%). Image analysis was performed using Fiji version 2.0 [56]. Mitochondrial and Golgi morphology analysis was performed by computing the number of cells displaying different morphological features and determining the statistical significance of the differences using Chi-square test and Bonferroni correction as described below.

### 2.10. Statistical Analysis

Statistical significance of the differences between means was calculated using one-way ANOVA and Dunnet´s or Tukey´s multiple comparisons *post-hoc* tests. Only biological replicates were considered for statistical analysis of the means. For frequency distribution analysis, absolute counts derived from more than one experiment were computed. In this case, Chi-square test and Bonferroni correction was used to determine statistical significance between control and individual conditions. The assumed type I error was α = 0.05 in all cases. Statistical methods and significance thresholds are described in the corresponding figure legends. Data were processed using Excel (Microsoft, Redmond, WA, USA) and GraphPad Prism (San Diego, CA, USA).

## 3. Results

### 3.1. Lipin2 Silencing Interferes with HCV Propagation

**Figure 1:**
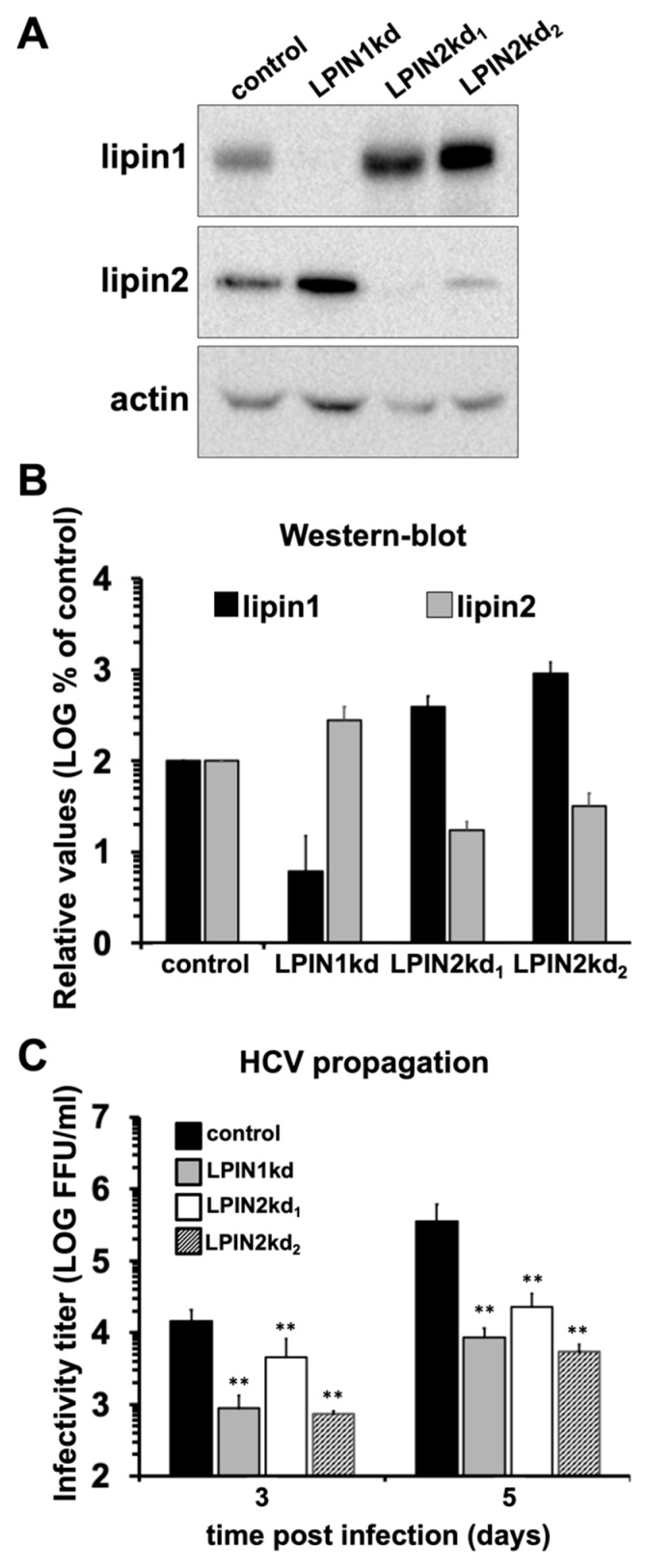
Lipin2 silencing interferes with HCV propagation. Huh-7 cells were transduced with lentiviral vectors expressing shRNAs targeting LPIN1 (LPIN1kd) or LPIN2 (LPIN2kd_1_ and LPIN2kd_2_). A control cell line was generated in parallel using a lentiviral vector expressing a non-targeting shRNA (control). Seven days post-transduction, cell lysates were subjected to Western-Blot analysis using antibodies against lipin1, lipin2 or tubulin as loading control. (**A**) Representative images showing lipin1 and lipin2 accumulation in the different cell lines at day 7 post-transduction. (**B**) Quantitation of lipin1 and lipin2 accumulation in the different cell lines. Data are shown as average and SD of four independent experiments (*n* = 4). Time 0 displays the limit of detection in this assay. (**C**) Control and lipin-deficient cells were inoculated with HCV (D183v) at MOI 0.01 and cultured for 5 days. Extracellular infectivity titers were determined in cell supernatants collected at day 3 and 5. Data are shown as average and SD of nine biological replicates from three independent experiments (*n* = 9). Statistical significance was evaluated using one-way ANOVA and Dunnet´s *post hoc* test. (** *p* < 0.01).

We recently reported that lipin1 plays a specific role in early stages of HCV infection [29]. Given that lipin1 and lipin2 cooperate to maintain liver glycerolipid homeostasis, we set out to determine if lipin2 also plays a role in HCV infection. To generate lipin-deficient cultures, Huh-7 cells were transduced with lentiviral vectors expressing either an irrelevant shRNA (control), a LPIN1-targeting shRNA (LPIN1kd) as control or two different shRNAs targeting LPIN2 (LPIN2kd_1_ and LPIN2kd_2_). Lipin silencing was verified by western blot at day 7 post-transduction (Figure 1A,B). Lipin1 expression was reduced by approximately 10-fold in LPIN1kd cells with a concomitant 3-fold increase in lipin2 accumulation (Figure 1A,B). Lipin2 expression was reduced by more than 5-fold in LPIN2kd_1_ and 3-fold in LPIN2kd_2_ cells as compared with the controls (Figure 1A,B). Conversely, lipin1 protein was strongly accumulated in lipin2-deficient cells, with a 3-fold induction in LPIN2kd_1_ cells and by more than 6-fold in the LPIN2kd_2_ (Figure 1A,B).

To determine the impact of lipin2 silencing on HCV infection, we performed multiple cycle infection experiments in control and lipin2-deficient cells. We included LPIN1kd cells, which are partially refractory to HCV infection (Figure 1C and [29]), as control for comparison with LPIN2kd cultures. Multiple cycle infection experiments were carried by inoculating the control and knock-down cell cultures with HCV (genotype 2a D183v strain; MOI 0.01) and showed a reduction of more than two orders of magnitude in extracellular infectivity titers at day 5 post infection in both lipin1 and lipin2-deficient cell cultures (Figure 1C), suggesting that, similarly to lipin1, lipin2 is limiting for HCV propagation in cell culture.

### 3.2. Impact of Lipin2 Silencing on Early Aspects of HCV Infection

**Figure 2:**
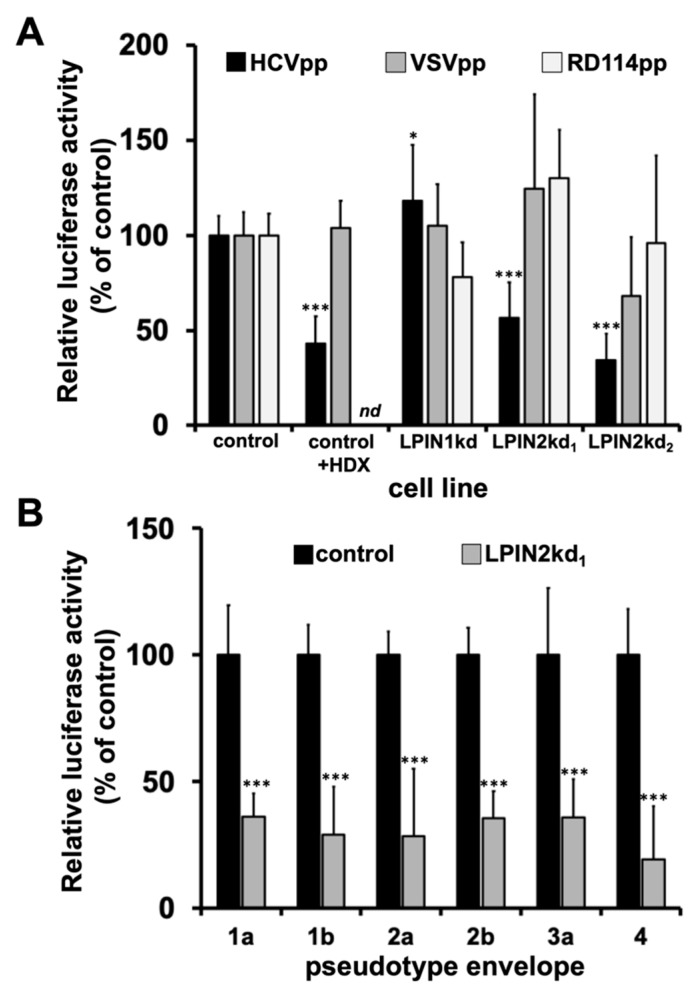

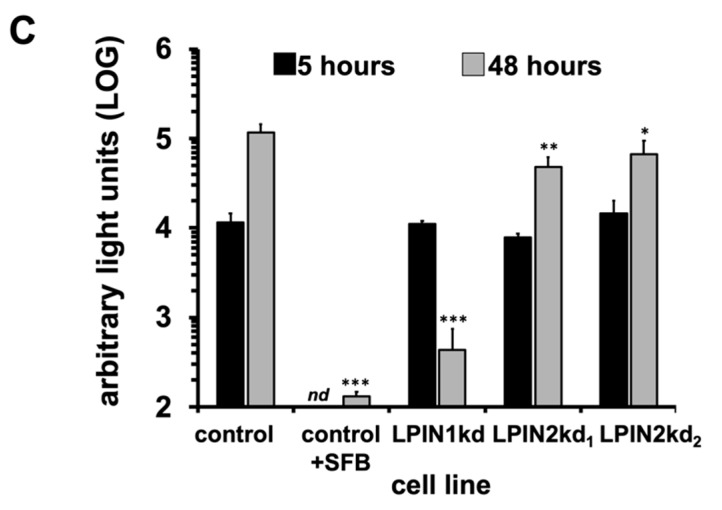
Study of early stages of HCV infection in lipin2-deficient cells. Huh-7 cells were transduced with lentiviral vectors expressing shRNAs targeting LPIN1 (LPIN1kd) or LPIN2 (LPIN2kd_1_ and LPIN2kd_2_). A control cell line was generated in parallel using a lentiviral vector expressing a non-targeting shRNA (control). At day 7 post transduction, the different cultures were inoculated with recombinant retroviral pseudotypes bearing (**A**) HCV (HCV*pp*); VSV (VSV*pp*) or RD114 (RD114pp) glycoproteins or (**B**) representative envelopes of major HCV genotypes. Intracellular reporter gene (luciferase) activity was determined forty-eight hours post inoculation. As control of HCV entry inhibition, control cells were treated with HCV entry inhibitor hydroxyzine pamoate (control+HDX; 5 µM). Normalized entry efficiency is shown as average and SD of a minimum of nine biological replicates from three independent experiments (*n* = 9). (**C**) At day 7 post transduction, the different cell lines were transfected with a dicistronic subgenomic replicon bearing a luciferase gene. Intracellular reporter gene (luciferase) activity was determined 5 and 48 h post inoculation in the replication-competent construct. Data are shown as average and SD of three biological replicates from a representative experiment. Statistical significance was evaluated using one-way ANOVA and Dunnet´s *post hoc* test. (* *p* < 0.05; ** *p* < 0.01; *** *p* < 0.001).

Given the impact of lipin2 silencing on HCV propagation, we set out to study at which level lipin2 was limiting for HCV infection using surrogate models of different steps of HCV replication cycle. We first studied viral entry using reporter retroviral particles, pseudotyped with viral envelopes corresponding to HCV (HCVpp), vesicular stomatitis virus (VSVpp) [60,68] or an endogenous feline retrovirus (RD114pp) [62]. Treatment of the cells with hydroxyzine (5µM), a previously described HCV entry inhibitor [63], caused a significant 2-fold reduction of HCVpp but not VSVpp entry (Figure 2A; control+HDX), underscoring the notion that reporter luciferase activity indeed represents viral entry efficiency. As expected from our previous study, infection of lipin-1 deficient cells with HCV or VSVpp resulted in normal entry (Figure 2A) [29]. In contrast, lipin2 silencing significantly reduced HCVpp entry in both LPIN2kd1 (56 ± 18%) and LPIN2kd2 cells (34 ± 14%; Figure 2A). However, this effect appears to be specific for HCV, since neither VSVpp nor RD114 infection efficiency is significantly reduced in lipin2-deficient cells (Figure 2A). Infection of control and LPIN2kd_1_ with HCVpp from major genotypes confirmed that lipin2 silencing interferes similarly with entry of all tested HCV genotypes (Figure 2B). These results suggest that lipin2 silencing interferes with entry of HCVpp from major HCV genotypes.

Next, we set out to determine if lipin2 silencing interferes with aspects of the infection downstream of viral entry. To bypass entry, an in vitro transcribed RNA corresponding to a subgenomic replicon bearing a luciferase gene was transfected into control and lipin2-deficient cells. Cells were harvested at 5 and 48 h post-transfection to determine relative luciferase activity as readout of direct translation of the incoming RNAs (primary translation; 5 h) or HCV RNA replication efficiency (48 h). Control and lipin2-deficient cells display comparable levels of luciferase 5 h post-transfection, indicating that transfection efficiency as well as primary translation was not altered by lipin2 silencing (Figure 2C). Luciferase activity measured at 48 h was reduced by three orders of magnitude in the presence of sofosbuvir (SFB; 1 µM), a potent HCV RNA replication inhibitor, demonstrating that luciferase activity represents replication efficiency in this experimental setup (Figure 2C; control+SFB). As expected, LPIN1kd cells showed a reduction of more than two orders of magnitude in RNA replication as previously reported [29]. In contrast with the strong defect observed in LPIN1kd cells, replication efficiency was only reduced by approximately 2-fold in lipin2-deficient cells (LPIN2kd_1_ 41 ± 11%, LPIN2kd_2_ cells 57 ± 23%; Figure 2C), suggesting that lipin2 silencing also interferes with HCV RNA replication in this surrogate model of infection, although with substantially lower magnitude than in lipin1-deficient cells.

### 3.3. Lipin2 Interferes with Virion Secretion During HCV Infection

**Figure 3:**
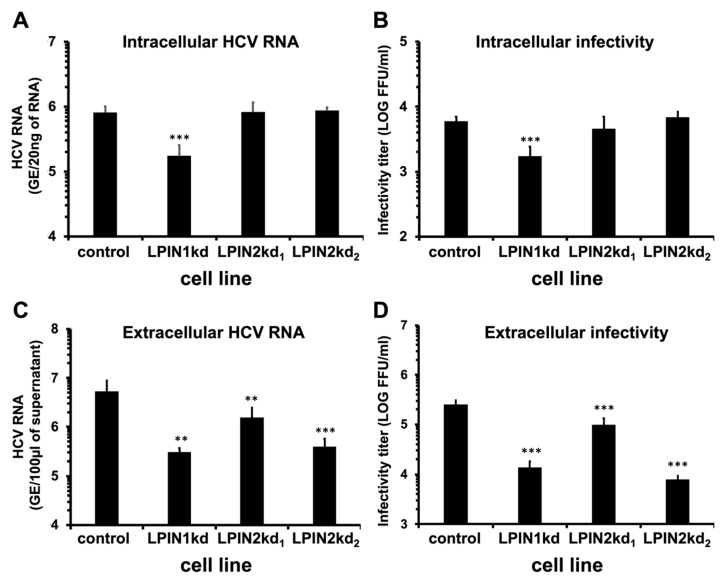
Lipin2 silencing interferes with late stages of HCV infection. Huh-7 cells were transduced with lentiviral vectors expressing shRNAs targeting LPIN1 (LPIN1kd) or LPIN2 (LPIN2kd_1_ and LPIN2kd_2_). A control cell line was generated in parallel using a lentiviral vector expressing a non-targeting shRNA (control). At day 7 post transduction, the different cell lines were inoculated with HCV (D183v) at MOI 10. Samples of the cells and supernatants were collected 48 h post-transduction to determine HCV RNA levels by RT-qPCR or viral infectivity titers by endpoint dilution. (**A**) Intracellular HCV RNA levels. (**B**) Intracellular infectivity. (**C**) Extracellular infectivity. (**D**) Extracellular HCV RNA levels. Data are shown as average and SD of six biological replicates from two independent experiments (*n* = 6). Statistical significance was evaluated using one-way ANOVA and Dunnet´s *post hoc* test. (** *p* < 0.01; *** *p* < 0.001).

The relatively small impact of lipin2 silencing on early infection events (Figure 2) contrasts with a strong reduction in virus propagation shown in multiple cycle infection experiments (Figure 1). To evaluate the role of lipin2 at late stages of the infection, control, lipin1 or lipin2-deficient cells were inoculated at MOI 10 with HCV (D183v strain; genotype 2a) and samples of the cells and supernatants were collected at 48 h post-inoculation. Consistent with our previous report [29], LPIN1kd cells showed a significant reduction in the accumulation of HCV RNA (Figure 3), intracellular and extracellular infectious particles (Figure 3), confirming that lipin1 silencing interferes with early stages of HCV infection leading to viral RNA accumulation. In contrast, LPIN2kd cells display relatively normal HCV RNA as compared with control cells (Figure 3A), supporting the notion that early stages of the infection are marginally affected in the context of a *bona fide* infection performed at high multiplicity. Intracellular infectivity titers were determined to evaluate infectious virus assembly in the different cell lines. Intracellular infectivity titers were comparable in control and LPIN2kd cells, suggesting that infectious virus assembly is not affected by reduced lipin2 expression (Figure 3B). In contrast, extracellular infectivity titers as well as extracellular HCV RNA levels were significantly reduced by two-fold in LPIN2kd1 and by 30-fold in LPIN2kd2 cells (Figure 3C,D), suggesting that lipin2 silencing interferes with trafficking and/or release of infectious HCV virions. These results support the notion for a differential impact of lipin1 or lipin2 silencing at different stages of the HCV replication cycle, confirming a role for lipin1 at the onset of HCV RNA replication [29] and revealing a new role for lipin2 on infectious virus trafficking and release.

### 3.4. Lipin2 Silencing Causes Golgi Apparatus Fragmentation

**Figure 4:**
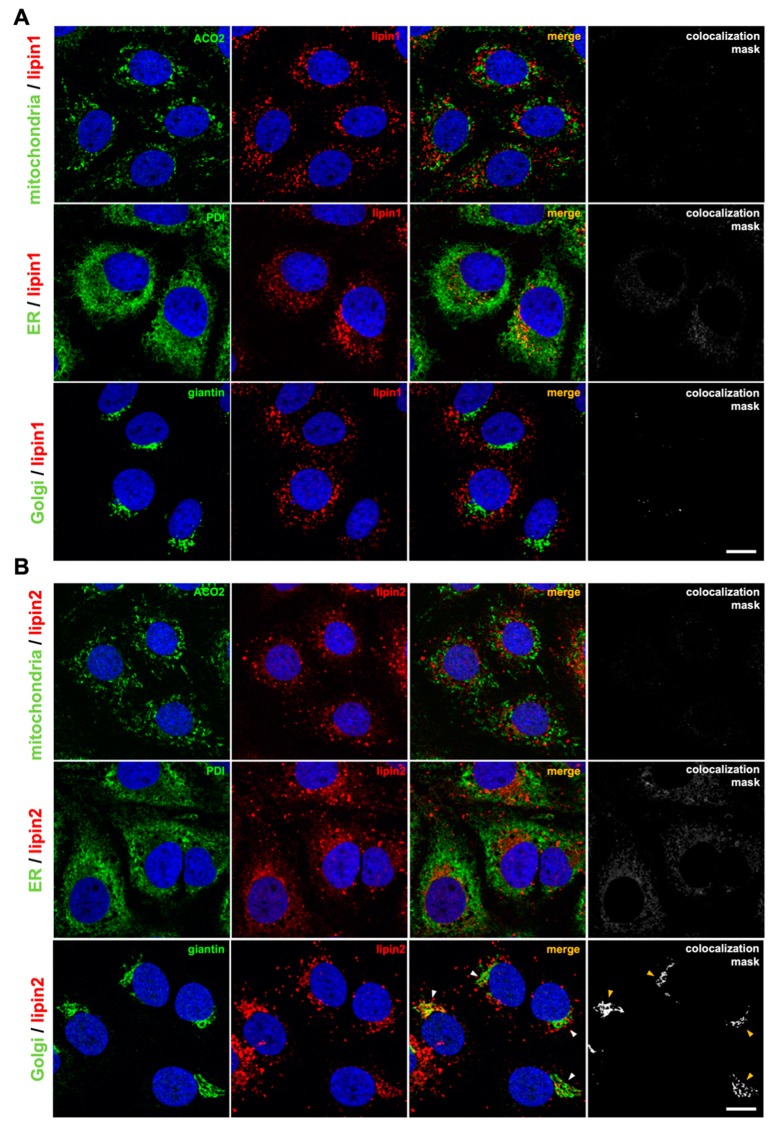
Lipin2 partially overlaps with subcellular Golgi distribution. Huh-7 cells were fixed with 4% formaldehyde and permeabilized using TX 100 as described in Materials and Methods. Subcellular localization of (**A**) lipin1 (in red) or (**B**) lipin2 (in red) was analyzed by colocalization analysis with markers (in green) of ER (PDI), mitochondria (ACO2) or Golgi (giantin) by immunofluorescence confocal microscopy. Representative images show lipin1 or lipin2 (red) with the different organelle markers (green). Nuclei were stained with DAPI and are shown in blue. Colocalization masks were generated using Fiji software. Scale bar 20 microns.

Lipin1 has previously been involved in endo-lysosomal trafficking [37], and Golgi maintenance [69]. In order to gain insight into the differential cellular roles of lipins, we analyzed lipin1 and lipin2 subcellular localization using specific antibodies to determine colocalization with Golgi (giantin), ER (PDI) and mitochondria (ACO_2_) using confocal immunofluorescence microscopy in uninfected Huh-7 cells (Figure 4).

**Figure 5:**
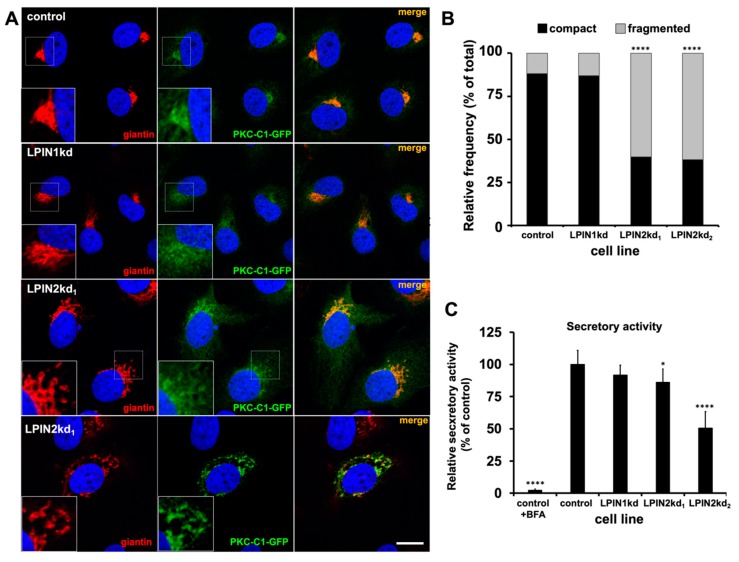
Lipin2, but not lipin1, silencing causes morphological alterations of the Golgi apparatus. Huh-7 cells constitutively expressing a DAG sensing probe (PKC-C1-D1-GFP) (see Materials and methods) were transduced with lentiviral vectors expressing non-targeting (control), shRNAs targeting LPIN1 (LPIN1kd) or LPIN2 mRNA (LPIN2kd_1_ and LPIN2kd_2_). At day 7 post transduction, control and lipin-deficient cultures expressing the DAG probe were fixed and processed for immunofluorescence microscopy using antibodies against a Golgi Apparatus marker (giantin) (**A**) Representative images of the Golgi morphology (red) and DAG probe (green) PKC-C1-GFP different cell lines. Nuclei were stained with DAPI and are shown in blue. (**B**) Relative frequency of the different Golgi morphologies. Data from four independent experiments are shown as percentage of the number of cells displaying compact or fragmented Golgi per total number of cells. Statistical significance was evaluated using Chi-square test and Bonferroni correction (n_control_ = 868, n_LPIN1kd_ = 578, n_LPIN2kd1_ = 633, n_LPIN2kd2_=568; * *p* < 0.0083; **** *p* < 0.0001). (**C**) Relative secretory capacity of lipin-deficient cells as determined by overexpression and secretion of Gaussia luciferase. Control cells were treated with secretion inhibitor brefeldin A (1 µg/mL; control+BFA). Data are shown as average and SD of eight biological replicates from two independent experiments (*n* = 8). Statistical significance was evaluated using one-way ANOVA and Dunnet´s *post hoc* test. (* *p* < 0.05; **** *p* < 0.001).

Lipin1 staining appeared as cytoplasmic *punctae* that did not significantly colocalize with any of these membranous compartment markers (Pearson´s < 0.5; Figure 4). Lipin2 staining showed two differentiated patterns: a relatively dim perinuclear signal and bright, cytoplasmic particles. Lipin2 did not significantly colocalize neither with mitochondrial nor ER markers (Pearson´s < 0.5; Figure 4). Although the overall lipin2 and Golgi marker giantin signals do not strictly colocalize (Pearson´s = 0.34 ± 0.08), perinuclear diffuse lipin2 staining overlapped with giantin (Figure 4; arrowheads in merge and colocalization mask). These results suggest that, in contrast to lipin1 (Pearson´s = 0.05 ± 0.02; Figure 5), a fraction of lipin2 colocalizes with Golgi apparatus markers.

Given lipin2 subcellular localization and the interference of lipin2 silencing with HCV secretion, we sought to evaluate the regulatory function of lipin2 on the Golgi-localized DAG pool and/or on Golgi morphology. To this aim, we used the domain C1 of Protein kinase C, a sensor for the DAG known to localize in Golgi [70], combined with detection of the Golgi marker giantin. Lipin1 silencing did not significantly alter neither giantin localization nor DAG-probe subcellular localization (Figure 5A). In contrast, lipin2 silencing caused a clear increase in the proportion of cells with a fragmented Golgi apparatus, coinciding with a redistribution of the intracellular DAG probe (Figure 5A). The increase in Golgi apparatus fragmentation frequency was statistically significant in LPIN2kd_1_ and LPIN2kd_2_ cells and was not observed in lipin1-deficient cells and (Figure 5B). These results suggest that lipin2, but not lipin1, silencing causes Golgi apparatus fragmentation and alteration of intracellular DAG pool distribution. To determine the functional impact of these morphological alterations on overall Golgi function, we measured the secretory capacity of the different cell lines by transfecting a plasmid encoding Gaussia luciferase, an enzyme that is efficiently secreted to the cell supernatant. Analysis of the ratio secreted/intracellular Gaussia luciferase activity in the uninfected cell supernatants and lysates indicates that LPIN1kd secretory capacity is comparable to that of the control, in contrast with the strong reduction of the ratio observed when treating the cells with brefeldin A, a broadly used secretion inhibitor [67] (Figure 5C). However, lipin2-deficient cells display a significant reduction in their respective relative secretion capacity (82 ± 7% of the control in LPIN2kd_1_ and 53 ± 15% in LPIN2kd) suggesting that lipin2 silencing may moderately interfere with this cellular function (Figure 5C). Overall, this data show that lipin2 silencing selectively causes Golgi apparatus fragmentation, which may partially interfere with cell secretory capacity, supporting the notion that lipin2 participates in the maintenance of the structure of this subcellular compartment, a function that cannot be compensated by enhanced lipin1 accumulation.

### 3.5. Lipin2 Silencing Causes Mitochondria Elongation

**Figure 6:**
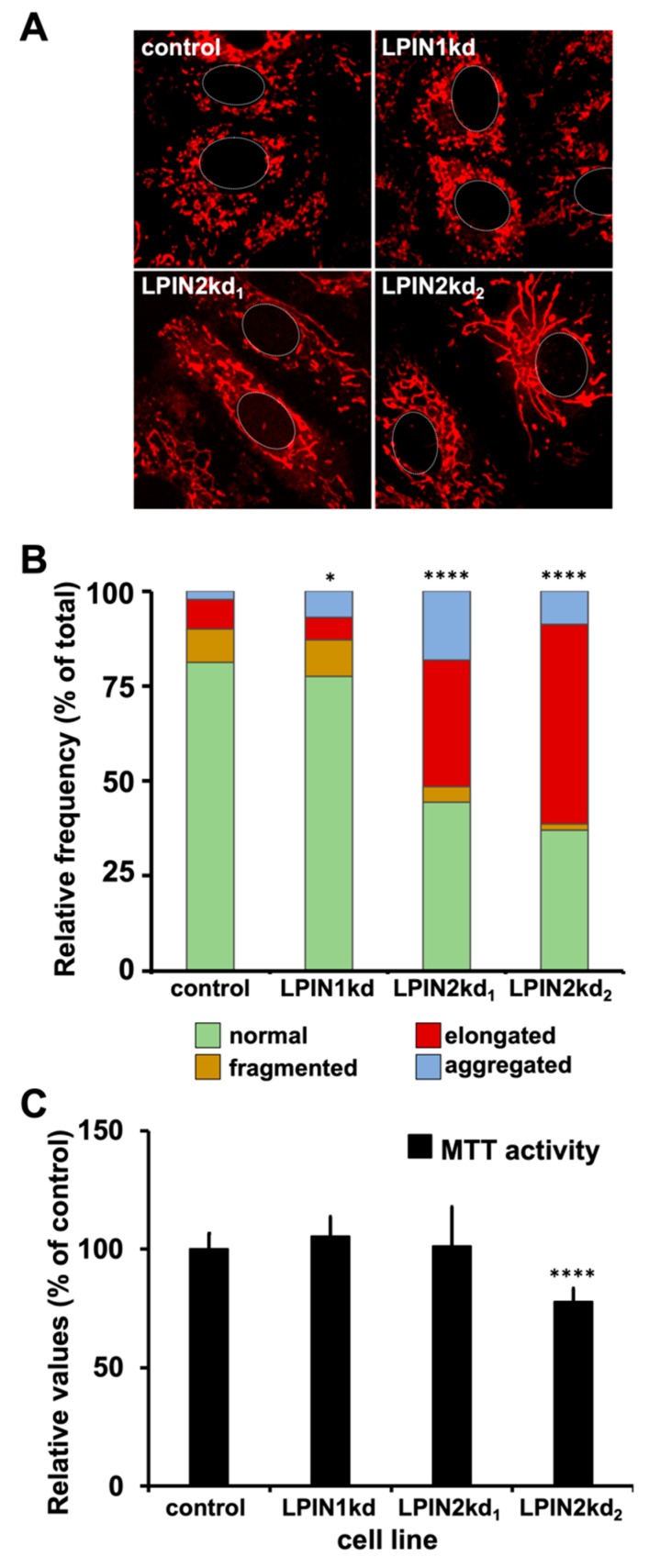
Lipin2 silencing causes mitochondrial elongation. Control, LPIN1kd, LPIN2kd_1_ and LPIN2kd_2_ cell cultures were stained with Mitotracker^TM^ red following manufacturer recommendations (Life Technologies) and imaged in vivo under a confocal microscope at 37 °C and 5% CO_2_ to visualize mitochondria. (**A**) Representative images of the mitochondrial morphology in the different cell populations. Cell nucleus is approximately delimited by a dotted white line for reference. (**B**) Relative frequency of the different mitochondrial morphologies in the analyzed cell cultures. Data from two independent experiments are shown as percentage of the number of cells displaying the different mitochondrial morphologies per total number of cells. Statistical significance was evaluated using Chi-square test and Bonferroni correction (n_control_ = 475, n_LPIN1kd_ = 581, n_LPIN2kd1_ = 410, n_LPIN2kd2_ = 251; * *p* < 0.0083; **** *p* < 0.0001). (**C**) Estimation of mitochondrial activity by an MTT assay using six biological replicates from two independent experiments (*n* = 6). Statistical significance was evaluated using one-way ANOVA and Dunnet´s *post hoc* test. (* *p* < 05; **** *p* < 0.001).

It has previously been shown that lipin1 participates in the regulation of mitochondrial dynamics by generating DAG at the mitochondrial surface and recruiting the mitochondrial fission machinery [31]. Since the role of lipin2 on mitochondrial dynamics has not been previously documented, we sought to determine the impact of lipin2-silencing on mitochondrial morphology. Control, LPIN1kd, LPIN2kd_1_ and LPIN2kd_2_ cells were stained with Mitotracker^TM^, followed by live imaging using confocal microscopy. Inspection of individual control Huh-7 cells revealed a majority of cells displaying mostly rod-shaped, relatively short mitochondria, with a minority of cells displaying fragmented or aggregated mitochondria (Figure 6; control). Similar mitochondrial frequencies were found in LPIN1kd cells, with a slight but statistically significant increase in the number of cells displaying mitochondrial aggregates (Figure 6A,B). Lipin2-deficient cells also displayed a significant increase in the frequency of cells with fragmented or aggregated mitochondria (Figure 6A,B). However, the most striking difference in lipin2-deficient cell populations was a significant enrichment in cells with elongated mitochondria, the frequency of which increase from 5–8% in control and lipin1-deficient cells to 35% in LPIN2kd_1_ and above 50% in LPIN2kd_2_ cells (Figure 6B). Similar results were obtained when silencing lipin2 in a cell line expressing YFP-tagged mitochondria (data not shown).

These results suggest that lipin2 silencing causes alterations in mitochondrial fusion/fission dynamics, but only partially altering overall cellular mitochondrial activity in the case of LPIN2kd_2_ cells, as determined by an MTT assay (Figure 6C). Interestingly, similar mitochondrial elongation events have been reported in NIH3T3 cells after lipin1 silencing [31], underscoring the role of lipins in mitochondrial dynamics while suggesting that different lipin genes may play the leading role in different cell lines.

### 3.6. Lipin2, but not Lipin1, Silencing Interferes with Flavivirus Spread

**Figure 7:**
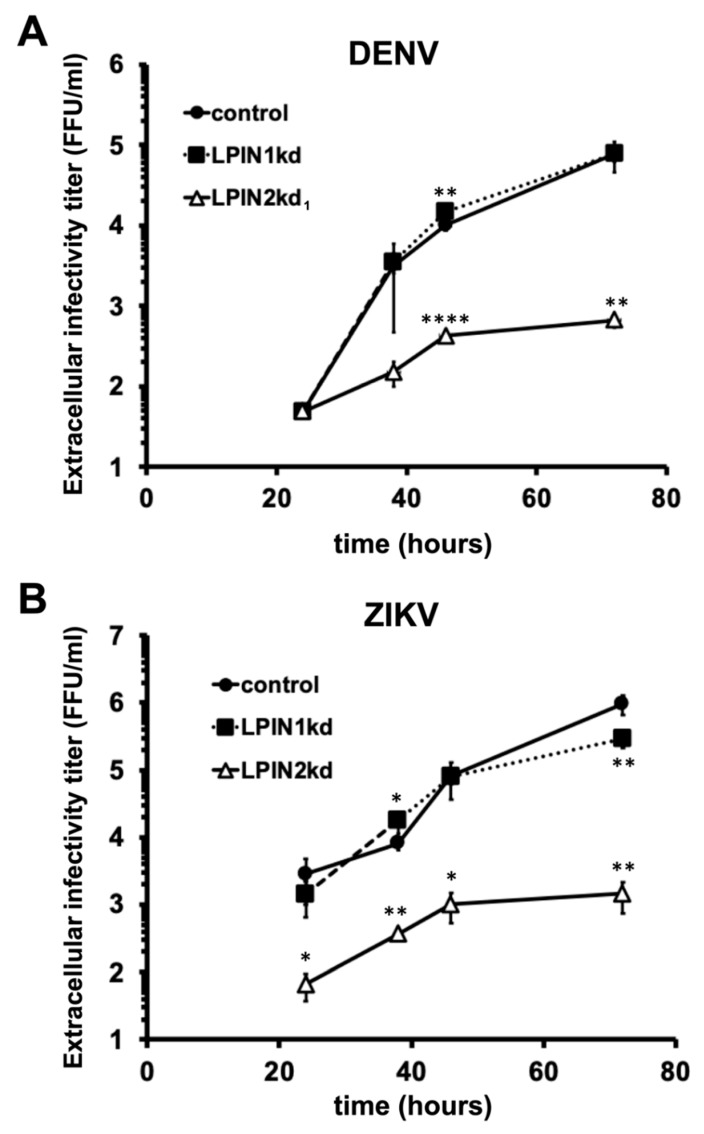
Lipin2, but not lipin1, silencing interferes with DENV and ZIKV virus propagation. Huh-7 cells were transduced with lentiviral vectors expressing non-targeting (control), shRNAs targeting LPIN1 (LPIN1kd) or LPIN2 mRNA (LPIN2kd_1_ and LPIN2kd_2_). At day 7 post transduction, the different cultures were inoculated with DENV (NGC strain), with ZIKV (BeH819015 strain). Samples of the cell supernatants collected at the indicated time points were used to determine extracellular infectivity titers. (**A**) DENV and (**B**) ZIKV extracellular infectivity titers. Data are shown as average and SD of three biological replicates (*n* = 3) from one experiment. Statistical significance was evaluated using one-way ANOVA and Dunnet´s *post hoc* test. (* *p* < 0.05; ** *p* < 0.01; **** *p* < 0.001).

The aforementioned data suggest a role for lipin2 in the maintenance of mitochondria and Golgi apparatus structure, and the alterations observed in lipin2-deficient cells could interfere with replication of other viruses. Thus, we set out to determine the impact of lipin2 silencing on the spread of other *Flaviviridae*. Infection of control, lipin1- and lipin2-deficient Huh-7 cells with dengue (DENV; NGC strain) or Zika virus (ZIKV; BeH819015 strain) at MOI 0.01 resulted in a reduction of more than two orders of magnitude in LPIN2kd_1_ cells as compared with control cells at different times post infection (Figure 7), suggesting that lipin2 silencing interferes not only with HCV, but also with other members of the *Flaviviridae* family. This situation is in clear contrast with lipin1-deficient cells, where DENV and ZIKV efficiently propagate (Figure 7). 

These results suggest that, in contrast to lipin1, cellular alterations induced by lipin2 silencing limit replication by all tested viruses, underscoring the notion that lipin1 and lipin2 may play different roles in Huh-7 cells. This is further supported by the fact that increased lipin1 accumulation could not functionally compensate lipin2 deficiency to support HCV infection and vice versa (Figure 1) and [29].

## 4. Discussion

In this study, we have determined important functional differences between lipin1 and lipin2 in the HCV replication cycle. While lipin1 is rate limiting for the formation of membranous compartments hosting functional replicase complexes [29], lipin2 silencing limits the release of infectious virions to the extracellular milieu (Figure 3C,D). In contrast with the selective impact of lipin1 silencing on HCV propagation, lipin2 silencing interfered with the propagation of other positive-strand RNA viruses (DENV, ZIKV; Figure 7), suggesting a more general mechanism of inhibition or a pleiotropic impact of lipin2 silencing on different aspects of cell homeostasis. In view of the impact of lipin2 silencing with Golgi apparatus and mitochondrial morphology (Figure 5 and Figure 6), it is possible that the mechanisms by which lipin2 silencing interferes with propagation of the different viruses is different in each case.

In the context of *bona fide* HCV infection, lipin2 silencing only significantly interfered with virus release in single cycle infection experiments (Figure 3). Normal intracellular HCV RNA accumulation in this context indicates that early stages of the replication cycle leading to viral RNA and intracellular infectious virion accumulation, including viral entry, primary translation, RNA replication and intracellular virion assembly are not significantly altered by lipin2 silencing (Figure 3A). These data somehow conflict with data collected using surrogate models of infection and reporter gene readouts, that suggest partial inhibition of HCVpp entry and subgenomic replicon RNA replication (Figure 2). Although statistically significant, the differences observed in Figure 2A–C may be not be biologically relevant in the context of a genuine HCV infection, since they are not reflected in reduced accumulation of the viral RNA in single cycle infection experiments (Figure 3A), which is expected if viral entry and HCV RNA replication are significantly affected. While the data obtained with HCVpp may reflect interference of lipin2 silencing with HCVpp entry (Figure 2A,B), they also reflect that HCVpp and *bona fide* HCV virion properties are not identical [71]. Thus, our data support the notion that limited virus release is the main contributor for the reduced virus propagation observed in multiple cycle infection experiments.

The cellular and molecular processes underlying HCV virion release are still not completely understood. Given that HCV virions are released in a non-cytolytic manner and that they resemble VLDL, it was originally assumed that they followed a relatively canonical secretory pathway involving Golgi apparatus [26,58]. However, compelling evidence suggests that HCV is released by a non-canonical secretion route that differs from that of host lipoproteins [21,72,73]. In fact, although host factors involved in trafficking through Golgi apparatus have been shown to be essential for HCV release [22,73], Golgi apparatus morphology is altered during HCV infection [74]. Thus, it is possible that, similarly to other positive-strand RNA viruses [75,76], HCV interferes with canonical secretory routes by altering intracellular lipid gradients, to optimize not only the generation of replication and assembly organelles, but also for efficient virion release [10,76,77].

Pharmacological interference with endosomal dynamics as well as silencing of components of the endosomal machinery inhibit HCV release but not VLDL secretion [21,23,24,72,78,79]. Moreover, silencing of clathrin and clathrin-adaptor molecules interferes with release of infectious HCV [25,72] further reinforcing the involvement of endosomal components in HCV release. This notion is further supported by the fact that core protein colocalizes with several endosomal markers at late times post-infection [24,72] and that core-positive vesicles traffic together with Rab11a-positive recycling endosomes or VAMP1-positive secretory vesicles [22], further suggesting that HCV virions traffic through endosomal vesicles before release. While not representing *bona fide* HCV entry, HCVpp infection efficiency is significantly reduced in lipin2-deficient cells (Figure 2A,B). While these observations somehow conflicts with the normal HCV RNA accumulation observed in single cycle infection experiments (Figure 3A), they are consistent with the notion that Golgi apparatus fragmentation may interfere with normal endosomal trafficking in lipin2-deficient cells, resulting in faulty HCV release.

Vesicular trafficking as well as membrane fusion and fission events are strongly determined by the presence and interconversion of PA and DAG in organellar membranes [69,80]. Local PA pools generated from membrane phospholipids by the action of different phospholipase D (PLD) enzymes [81] or by diacylglycerol kinases from DAG [82], have been shown to play a crucial role in vesicular trafficking as well as in membrane fusion and fission events [83,84]. Similarly, signaling DAG may be generated from different sources such as phospholipase C (PLC) enzymes [85] or by PA-phosphatases, including lipins [37,86].

Regarding the relevance of lipins in these processes, it has been recently shown that deletion of the yeast lipin homolog has a strong impact in SNARE protein-regulated membrane fusion events [87] and mammalian lipin1 actively participates in mitochondrial fission-fusion dynamics by regulating, together with PLD6 (mitoPLD), PA levels at the mitochondrial surface [31,88]. In fact, lipin1 silencing in NIH3T3 cells leads to PA accumulation at the mitochondrial surface and subsequent mitochondrial elongation, while lipin1 overexpression in HeLa cells induce mitochondrial fragmentation [31]. We have shown that lipin2-deficient cells display a significantly higher proportion of cells with elongated mitochondria as compared with control cells (Figure 6). While both studies reinforce the notion that lipins regulate mitochondrial dynamics, they point at the possibility that lipin2 may also regulate mitochondrial dynamics in cells of hepatic origin, where lipin2 is predominantly expressed [37]. Thus, it is possible that lipin1 and lipin2 cooperate in the maintenance of correct mitochondrial dynamics and that one or the other may be limiting depending on their relative abundance. However, in our model, lipin2-deficient cells express higher lipin1 levels than the control cells (Figure 1A,B) and this could not compensate for the putative lipin2 mitochondrial function (Figure 6), suggesting that, at least in Huh-7 cells, lipin2 contributes to managing the mitochondrial PA pool to regulate fission/fusion events.

Both DAG and PA are required for Golgi apparatus membrane tubule and vesicle formation [32,69]. Although the precise regulation of their abundance is dependent on different factors, pharmacological inhibition of lipin PAP by propranolol causes reversible inhibition of Golgi apparatus vesicle and tubule formation, suggesting a role for lipins in Golgi apparatus dynamics [89,90]. Our results suggest that, in Huh-7 cells, lipin2 also manages DAG pools that are relevant to Golgi apparatus morphology, since lipin2 silencing resulted in a significant increase in the frequency of cells displaying fragmented Golgi apparatus structures (Figure 5A,B) and a significant reduction in the overall cell secretion capacity as determined by *Gaussia* luciferase secretion studies (Figure 5C). These results reinforce the notion that lipin2 manages lipid pools that are relevant for vesicular trafficking and organelle fusion/fission events to a greater extent than lipin1, at least in Huh-7 cells. Loss of these lipin2 functions in these cells interfere both with HCVpp entry (Figure 2A,B) and, more prominently, with virus exocytosis, suggesting that lipin2 participates in the coordination of vesicular trafficking that leads to HCV virion release. Taken together, the data presented in this study and those described in our previous lipin1 study [29], suggest differential roles for lipin1 and lipin2 in liver cells, not only for supporting efficient HCV infection, but also at the level of basic cellular functions, reinforcing the notion that, while some degree of functional overlap exists between lipin1 and lipin2 in the liver, these proteins show divergent roles in cell biology. In fact, their different specific activity as well as their differential regulation by phosphorylation [39] or sumoylation [91], lead to suggesting that lipin2 is a constitutive PAP enzyme with low specific activity, while the high specific activity of lipin1 and the tight regulation of its PAP activity enables responsiveness to very specific metabolic needs [39]. The relatively low specificity of lipin2 interference with viral infection, in contrast with the specific role of lipin1 at early stages of HCV infection reinforces this notion.
